# Amateur Athletic Union (AAU) Accessibility: An Area Deprivation Index (ADI) Analysis of National Basketball Association (NBA) Players' Profiles

**DOI:** 10.1007/s12178-024-09908-9

**Published:** 2024-06-11

**Authors:** Brandon R. Ho, Joshua A. Valenzuela, Alexander R. Markes, Nirav K. Pandya

**Affiliations:** 1 School of Medicine, University of California San Francisco, 513 Parnassus Ave, San Francisco, CA 94143 USA; 2 College of Osteopathic Medicine, California Health Sciences University, Clovis, CA USA; 3 Department of Orthopaedic Surgery, University of California San Francisco, San Francisco, CA USA

**Keywords:** AAU Sports, Youth Sports, Inequity, Disparity, NBA Basketball, Area Deprivation Index

## Abstract

**Purpose of Review:**

Youth sports are increasingly shifting towards a “pay to play” model which has introduced financial barriers to participation. The Amateur Athletic Union (AAU) is the main organization for club basketball, serving as a platform where young athletes can compete beyond the recreational level. Outside the realm of athletes who have access to state-of-the-art facilities and top-tier coaching, the pathway to playing basketball at the next level may be predominantly available to those who can afford the considerable costs of AAU participation. The objective of this study is to determine the accessibility of AAU teams of active National Basketball Association (NBA) players through use of the Area Deprivation Index (ADI).

**Recent Findings:**

We identified 114 AAU teams with physical addresses for 250 (50%) currently active domestic NBA players. The State ADI of the high schools as well as national and state ADIs of prior AAU teams of active NBA players were significantly skewed toward lower ADI rankings (higher socioeconomic status) (p < 0.05). The mean distance between high school location and AAU location was 170 miles.

**Summary:**

Prior AAU teams of currently active NBA players are more frequently located in areas of higher socioeconomic status with nearly 50% being within the top 3rd lower state decile as measured by the area deprivation index. Similarly, we found the high schools these players attended, as a proxy for areas they grew up in, were also more frequently located in areas of higher socioeconomic status.

## Introduction

Basketball is one of the most common school-sponsored sports in America but less than 5% of high school athletes proceed to college basketball [1]. Even with this small percentage, many athletes, parents, and coaches organize their athletic activity (including early sport specialization) around this goal [2]. The Amateur Athletic Union (AAU) has long been established as a venue for players to showcase their skills and gain exposure to college coaches who could potentially recruit them [3]. Founded in 1888, AAU initially created common standards in the US for amateur sports, later shifting to provide sports programs for participants of all ages in the late 1970’s [4]. Currently, AAU has nearly 700,000 members and 150,000 volunteers across 41 sports programs and 55 U.S. districts [4]. Currently, one of the first steps for athletes and their families who support their goals of playing basketball at the next level is to join an AAU club.

AAU basketball provides a competitive environment in which multiple highly ranked players are competing. However, access to AAU basketball can be challenging for players and their families due to financial constraints, geographic location, and availability of teams. The shift from school-based programs to AAU teams has highlighted socioeconomic disparities in the participation of youth basketball; reflecting disparities seen in youth sports in general [5, 6••]. The mounting costs of equipment, club teams, and other financial barriers restrict certain athletes from being able to participate. Private sport clubs have ballooned to a 15-billion-dollar industry with 63% of parents paying $1200 to $6000 annually for their kids to participate in sports [7]. Additionally, while there are AAU teams located in 56 districts throughout the country[8], players living in rural or under-resourced communities may not have access to local programs.

The socioeconomic profile of an athlete's hometown can have a significant impact on their access to sports infrastructure, quality programs, and familial financial support [9, 10]. For athletes hoping to play in college and eventually in the NBA, the high cost of participating in AAU basketball teams may restrict athletes who come from under-resourced communities. With nearly every American-born NBA player participating in AAU at some point in their career[11••], playing on an AAU team has become a prerequisite for college and professional basketball recruitment [12]. To date, there is limited research that examines the accessibility of AAU basketball relative to geographic location, online presence, and socioeconomic factors.

Various tools for measuring socioeconomic status have been reported in the literature including insurance based, personal questionnaire-based, and zip code based [13, 14]. Recently there has been increased interest in the use of the Area Deprivation Index (ADI) which provides both state decile and national percentile rankings for the socioeconomic deprivation of neighborhoods based on the domains of income, education, employment, and housing quality. This database uses a locations physical address as opposed to postal zip codes for its rankings which may be more accurate for a given region’s socioeconomic status (SES) and has been previously used in orthopedic research evaluating disparities [14].

The financial and geographical barriers of participating in AAU teams may be contributing to the decreasing number of athletes from lower socioeconomic areas in college basketball rosters. Heightened academic requirements for college basketball recruitment, coupled with the high cost of early training presents substantial obstacles for athletes from undeserved areas. Recruited athletes with athletic scholarships are primarily middle-class students with college-educated parents, not those from low SES backgrounds who would benefit the most [15]. As one of the main recruiting platforms for high school players, the accessibility of AAU teams is instrumental in determining who gets to play basketball at the next level.

The objective of this study is to examine the socioeconomic profile of the prior AAU teams and high schools of active NBA players through use of the Area Deprivation Index. Our hypothesis is that both prior AAU teams and high schools that the players attended will be located in areas of higher socioeconomic status.

## Methods

### Data Source

The official National Basketball Association (NBA) website was queried to identify all active NBA rosters for the 2022–2023 season[16]. The youth basketball team and prior high school of all active NBA players were evaluated using the Circuit Scouting database, a company widely regarded for its wealth of information on player history and youth team demographics in the United States[11••]. If a player’s youth playing history or high school was not listed in the Circuit Scouting database, this information was obtained through searching various online outlets including but not limited to a school’s webpage, news outlets with sports webpages, AAU team webpages, and published information on social media accounts of players or teams. Variables obtained regarding player history included the player’s youth team name, league, physical address of most common practice site or headquarter location, website, and contact information.

To determine data on socioeconomic status (SES), the physical addresses of both the NBA players' respective high schools and AAU teams were used to calculate their Area of Deprivation Index (ADI). The Area of Deprivation Index is a validated source used to rank neighborhoods of interest on a state or federal level by socioeconomic disadvantage created by the Health Resources and Services Administration (HRSA) [17, 18]. The ADI ranking is formulated by and not limited to neighborhood income, education, employment, and housing quality data from the American Community Survey (ACS) Five-Year Estimates from 2015–2020. The percentiles are constructed by ranking the ADI from low to high for the nation and grouping the neighborhoods into percentile or decile bins. The state ADI is a decile ranking ranging from 1–10, while the national ADI is a percentile ranking ranging from 1–100. A ranking value of 1 in either ADI indicates the “least of disadvantaged neighborhood” (high SES), while a state ADI decile ranking of 10 or a national ADI percentile ranking of 100 is the “highest level of disadvantage” (low SES)[17, 18].

### Statistical Methods

Pearson Correlations were used to evaluate associations between AAU and high school state and national ADIs. Histogram plots of AAU and high school state and national ADIs were also generated and evaluated for normality via the Shapiro–Wilk test and skewness towards a certain socioeconomic status using the Skewness test and Kurtosis test. Skewness is a measure of the lack of symmetry while Kurtosis is a measure of whether the data are heavy-tailed or light-tailed relative to a normal distribution[19]. Kernel density estimates were added to the histograms to better visualize the distribution of data. Excess Kurtosis (p < 0.05) indicates tails of a histogram distribution are larger. In our example, a significant Kurtosis indicates more patients in the extremes of ADI. Straight line distance between AAU and high school addresses was also calculated. The difference between AAU and high school state and national ADIs, hereafter referred to as “delta value”, was analyzed. The mean and standard deviations for delta values of state and national ADIs were calculated and graphed. Straight line distances between AAU and high school addresses were also calculated via online software created by Map Developers[20]. The mean and standard deviation of the straight-line distances were performed and graphed. All analyses were performed using STATA, version 17 (StataCorp. 2021. *Stata Statistical Software: Release 17*. College Station, TX: StataCorp LLC.).

## Results

### Player and Team Demographics

590 active players entering the 2022–2023 NBA season were identified of which 500 (84.7%) were domestic players that played in AAU prior to their NBA careers. We identified 114 AAU teams with physical addresses for 250 (50%) currently active domestic NBA players. Of these 114 AAU teams, 70% (80) provided a website, 39% (45) a phone number, 67% (76) an email, and 89% (101) a social media account.

### AAU and High School ADIs

**Fig. 1:**
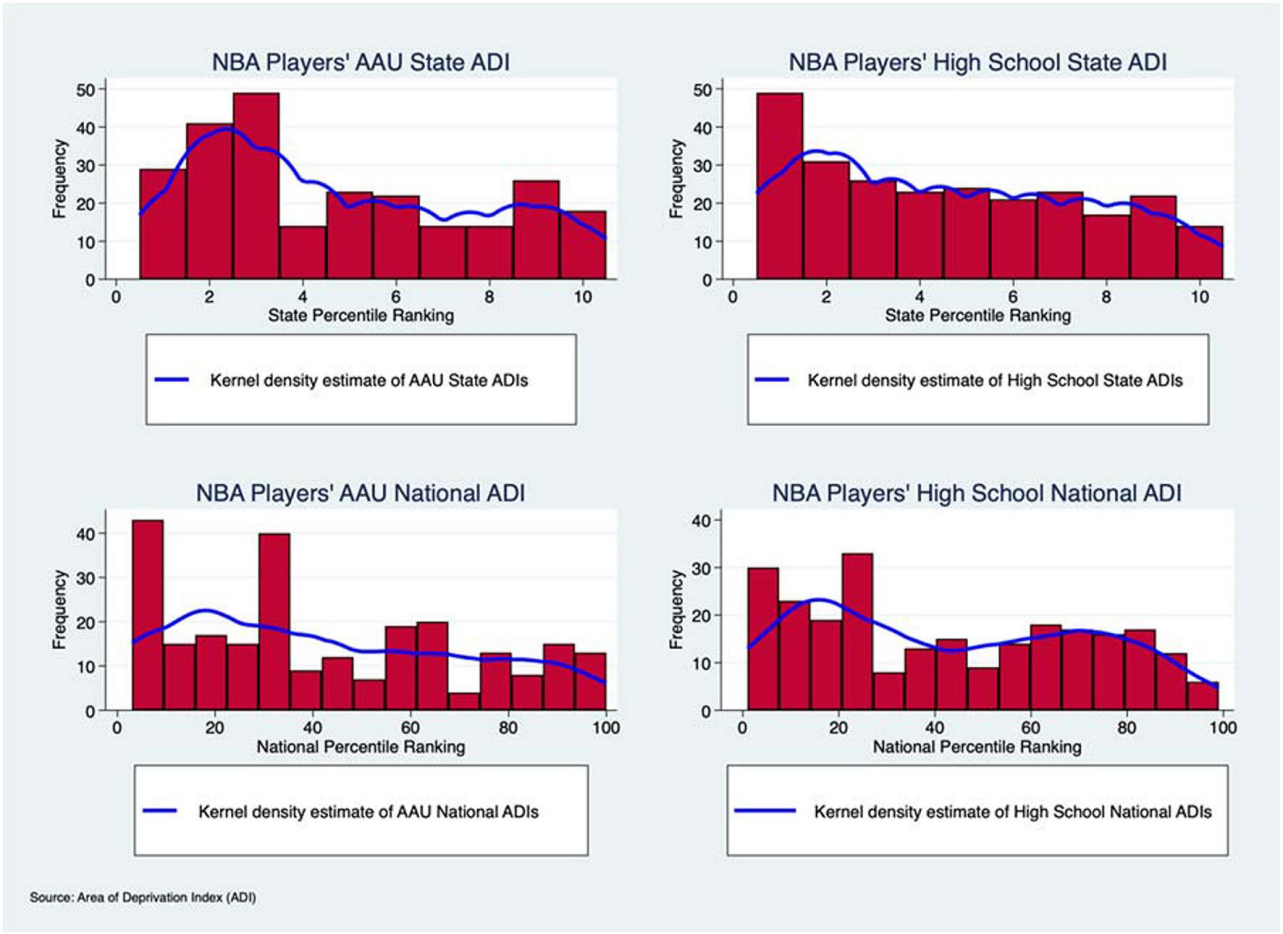
Side-by-side histogram comparisons and kernel density estimates of state and national Area of Deprivation Index scores for NBA player’s AAU and high school addresses

**Fig. 2:**
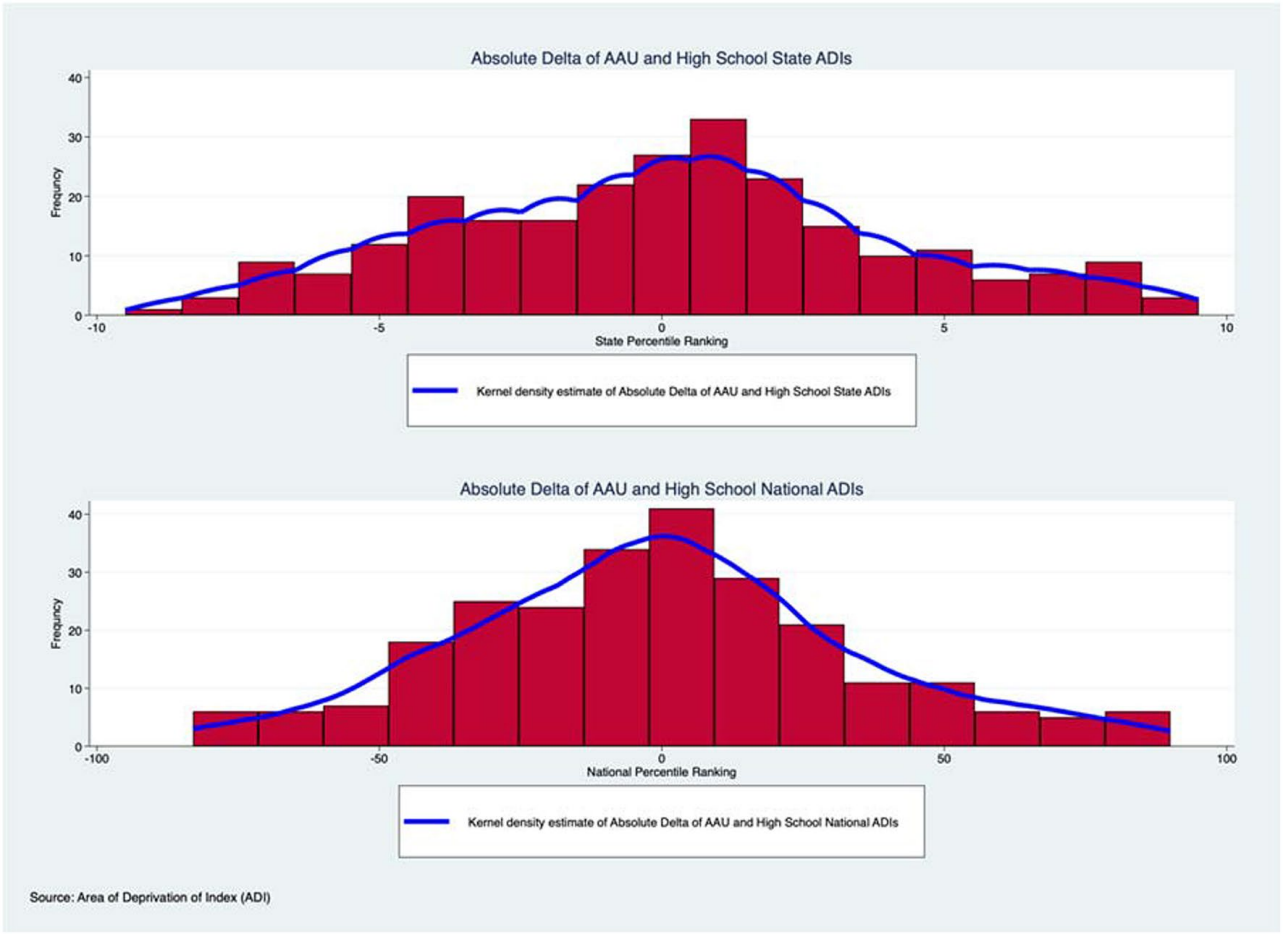
Histograms depicting the kernel density estimates and distribution of absolute delta values of state and national Area of Deprivation Index scores of NBA players’ AAU and high school addresses

Histogram plots of the respective AAU and high school ADIs are seen in Fig. 1 visually demonstrating relative normality or skewness. 42% of prior high schools that NBA players attended and 45% of AAU teams they played for were in the top 3rd decile for lower state ADI, corresponding to a higher SES. Results of the skewness testing of histogram plots for state and national ADIs of high schools and AAU teams are seen in Table 1. The state ADIs of the high schools were not normally distributed and significantly skewed toward lower ADI rankings or higher SES. (Shapiro–wilk, p < 0.01; Skewness test, p = 0.049; Kurtosis test, p < 0.01). The state and national ADI of the AAU team a player attended was not normally distributed and significantly skewed toward lower ADI rankings or higher SES (State: Shapiro–wilk, p < 0.01; Skewness test, p < 0.01; Kurtosis test, p < 0.01; National: Shapiro–wilk, p < 0.01; Skewness test, p = 0.0146; Kurtosis test, p < 0.01). The national ADI of the high school a player attended was not normally distributed though was also not significantly skewed (Shapiro–Wilk, p < 0.001; Skewness test P = 0.1543; Kurtosis test, p < 0.001). Significantly positive correlations were observed between the national ADI and state ADI of the high school NBA player (R^2^ = 0.83, p < 0.05), the national ADI and state ADI of the prior AAU team of a player (R^2^ = 0.82, p < 0.05), and national ADI of the high school and the national ADI of the AAU team of a player (R^2^ = 0.27, p < 0.05). Results of the mean absolute delta values for state and national ADIs of high schools and AAU teams are seen in Fig. 2. The mean absolute delta between the state ADI of the AAU team and the high school was 0.092 ± 3.95. The mean absolute delta between the national ADI of the AAU team and the high school was 0.6 ± 34.91. The mean distance between the high school location and AAU location was 170 miles ± 308.9 miles.

**Table 1:** Analysis of Normality and Skewness of National Basketball Association Players’ Amateur Athletic Union and High School Area of Deprivation Indexes

Variable	Skewness test for normality	Kurtosis test for normality	Shapiro–Wilk test for normality *p-*value	Skewness test for normality *p*-value	Kurtosis test for normality *p*-value
AAU State Decile	0.44	1.87	< 0.01	< 0.01	** < **0.01
AAU National Percentile	0.38	1.91	< 0.01	0.015	** < **0.01
High School State Decile	0.30	1.81	< 0.01	0.049	** < **0.01
High School National Percentile	0.22	1.69	< 0.01	0.15	** < **0.01

Histogram Analysis with Kernel Density Estimates of National Basketball Association Players’ Amateur Athletic Union and High School State and National Area of Deprivation Indexes

Histogram Analysis with Kernel Density Estimates of National Basketball Association Players’ Absolute Delta of Amateur Athletic Union and High School Area of Deprivation Indexes

## Discussion

There is limited empirical data on the accessibility of AAU basketball relative to geographic location, online presence, and socioeconomic factors. We found that the location of AAU teams and the prior high schools of players currently in the NBA were in areas with higher SES and that there were correlations between the ADIs of the high schools and that of the AAU teams.

The increase in private sports participation combined with single sport specialization has created a youth sports climate that unfortunately has led to barriers for participation. These barriers are intimately associated with socioeconomic disparities that many communities face. This could be due to various factors including access to sports infrastructure, time commitment, travel distances, and familial financial support [6••]. While basketball is one of the most popular sports in America with over 33% of parents reporting that their child played basketball on a regular basis in 2021, access to basketball is affected by pay-to-play systems [21•]. The total cost of play for participating in AAU basketball can range from $400 to $4000 depending on the team [22]. While the annual AAU Athlete Membership fees are generally affordable ($14.00 to $39.00), registration, team dues, insurance, out-of-town trips, and tournament fees make up the bulk of the cost [22, 23]. As a result, basketball has become one of the most expensive sports with parents paying an average annual amount of $1,002 [21•]. Over the past two decades, high school girls' basketball has lost 19% of its players while other top female sports like track and field (+ 10%) have grown [21•]. Although the reasons for this are multi-factorial, financial barriers must be considered. Female youth athletes from lower socioeconomic backgrounds have decreased sports participation rates compared to their more privileged peers, with low SES being linked to reduced parental support and increased participation barriers among girls [24].

In addition, geographical proximity to AAU teams must be considered as well; given both direct and indirect costs of transportations. The geographic location of the AAU team may not be reflective of the communities from which it draws its player’s from. Although high school is not a true proxy for players’ hometowns, the mean straight-line distance between AAU and high school locations of 170 miles reflects the trend of athletes moving or traveling far to participate in highly competitive AAU teams. Although skill level is one means by which players can be invited to join these teams, there is also the real logistical challenge of having the financial means to move or afford high weekly travel costs, requiring significant commitment from family. Travel is now the costliest feature in youth sports with parents spending $260 per sport annually [21•]. Financial barriers are reported at substantially higher rates among lower-income families with 42% of lower-income families citing high costs as a barrier to sports participation compared to 26% of middle- and higher-income families [25]. As a result, inequity in terms of access to AAU teams exist.

Some would argue that many AAU teams will “sponsor” players who may have limited financial means as cost is a substantial barrier for AAU basketball particularly as many NBA players participated in AAU teams without cost. Top-level players can compete in “shoe circuits,” exclusive AAU tournaments, leagues, and teams created by large shoe brands like Adidas, Nike, and Under Armour. Almost half of the players currently rostered in the NBA competed on a shoe circuit team [11••]. Participation in these shoe circuits teams is free of charge to their players and the team will cover hotel, and other related fees. In return, these companies establish connections with promising young players and garner brand loyalty early on. While shoe circuits may increase access to AAU basketball, players must play at the highest level to be noticed by these teams and most AAU players will never have a chance to participate in shoe circuits. Shoe circuit AAU teams are a small subset of AAU basketball teams and are extremely competitive and have limited spots.

Furthermore, beyond financial and geographic restraints, the ability to even be able to identify teams may be prohibitive for low-income families without reliable internet access. Finding and contacting AAU teams is mainly accomplished online through internet searches or the AAU website which includes a tool to find nearby AAU teams [26]. The presence of a website, social media account, and methods of contacting the teams is crucial for the accessibility of AAU teams. Of the 114 AAU teams included in the study, 70% provided a website, 39% a phone number, 67% an email, and 89% a social media account. Although most of the AAU teams had a website, a lack of internet access can prevent families from identifying potential AAU teams. Broadband adoption rates in the United States are 62% for households earning less than $20,000, with low-income communities typically having the lowest adoption rates [27]. Without the internet, access to AAU teams is limited to word of mouth or connections. AAU teams that are formed locally by parents and not through an organization are often exclusive, and the limited number of spots can make competition for spots on AAU teams intense. Some teams require tryouts or extensive scouting, and players who do not perform well or do not have connections to coaches or other players may not make the cut [8].

Families that are unfamiliar with the AAU pipeline or lack the resources to support their children are at a significant disadvantage. Fewer than 1 in 5 students playing Division 1 basketball come from families in which neither parent went to college [15]. In 2010, the NCAA began asking college athletes whether they are first generation students as part of its GOALS Study, which captures the background and experience of those playing sports in the NCAA [28]. In men’s basketball, the sport that used to have the highest percentage of first-generation students, the number plummeted from 28 to 19% while women’s basketball experienced a similar drop [28]. Similarly, the intersection of race, social class, and family structure creates unequal opportunities for individuals to enter the NBA with white athletes from low-income families 75% less likely to become NBA players [29, 30].

Youth physical activity is associated with immediate overall health benefits while establishing behavioral carry-over into adulthood [31, 32]. In the context of public health, youth sports participation can facilitate cognitive, social, emotional, and psychological development [33]. However, the U.S. Department of Health and Human Services noted that only 58% of youth participated in a sport in 2017 with decreasing participation among females, racial and ethnic minorities, and youth from households of low SES [34]. Moreover, recreational sport programs in under-resourced communities are disproportionately affected by social and structural inequalities. Schools in lower SES areas tend to have limited resources, fewer intramural activities, and a scarcity of exterior athletic facilities [35•]. Youth from rural communities have inadequate physical activity facilities compared with urban and suburban areas and less frequent physical activity overall [36]. Access to these recreational spaces is key to promoting youth sports participation. A study found an 84% increase in youth sports activity when school facilities were open publicly compared to a nearby community with closed schoolyards [37]. The limited availability of recreational options and the shift towards private, expensive alternatives like AAU basketball is detrimental youth physical activity. More needs to be done to increase the accessibility of AAU programs and promote a diverse range of sports and recreational activities, ensuring that children have options that cater to their individual interests, abilities, and developmental needs.

There are several limitations in our study. The ADI is limited insofar as it uses American Community Survey (ACS) Five Year Estimates in its construction. For example, the 2018 ADI uses the ACS data for 2018, which is a 5-year average of ACS data obtained from 2014–2018. All limitations of the source data will persist throughout the ADI—results are subject to the accuracy and errors contained within the American Community Survey data release. Using the location of player’s high school may be limited as a true proxy for the player’s hometown. Additionally, our dataset is drawn from only active NBA players and did not include the vast majority of AAU teams that exist nationwide. It is important to note that the data used for analysis was limited to 50% of currently active NBA players. This partial representation introduces potential bias, as it is uncertain whether the AAU teams and high schools of the omitted players were from higher or lower socioeconomic areas.

## Conclusions

Prior AAU teams of currently active NBA players are more frequently located in areas of higher socioeconomic status with nearly 50% being within the top 3rd decile for lower state deciles as measured by the area deprivation index. Similarly, we found the high schools these players attended, as a proxy for areas they grew up in, were also more frequently located in areas of higher socioeconomic status. Future prospective studies are needed to evaluate solutions to barriers of entry for players living in underserved areas.

## Data Availability

No datasets were generated or analysed during the current study.
